# Brain reactivity during aggressive response in women with premenstrual dysphoric disorder treated with a selective progesterone receptor modulator

**DOI:** 10.1038/s41386-021-01010-9

**Published:** 2021-04-29

**Authors:** Elisavet Kaltsouni, Patrick M. Fisher, Manon Dubol, Steinar Hustad, Rupert Lanzenberger, Vibe G. Frokjaer, Johan Wikström, Erika Comasco, Inger Sundström-Poromaa

**Affiliations:** 1 Department of Neuroscience, Science for Life Laboratory, Uppsala University, Uppsala, Sweden; 2 Neurobiology Research Unit, Department of Neurology, Copenhagen University Hospital Rigshospitalet-Blegdamsvej, Copenhagen, Denmark; 3 Department of Clinical Science, University of Bergen, Bergen, Norway; 4 Department of Psychiatry and Psychotherapy, Medical University of Vienna, Vienna, Austria; 5 Mental Health Services, Copenhagen, Capital Region of Denmark Denmark; 6 Department of Surgical Sciences, Radiology, Uppsala University, Uppsala, Sweden; 7 Department of Women’s and Children’s Health, Uppsala University, Uppsala, Sweden

**Keywords:** Neuroscience, Psychiatric disorders

## Abstract

Premenstrual dysphoric disorder (PMDD) is a psychiatric condition characterized by late luteal phase affective, cognitive, and physical impairment. The disorder causes significant suffering in about 5% of women in their reproductive age. Altered sensitivity of cognitive-affective brain circuits to progesterone and its downstream metabolite allopregnanolone is suggested to underlie PMDD symptomatology. Core mood symptoms include irritability and anger, with aggression being the behavioral outcome of these symptoms. The present study sought to investigate the neural correlates of reactive aggression during the premenstrual phase in women with PMDD, randomized to a selective progesterone receptor modulator (SPRM) or placebo. Self-reports on the Daily Record of Severity of Problems were used to assess PMDD symptoms and gonadal hormone levels were measured by liquid chromatography tandem mass spectrometry. Functional magnetic resonance imaging was performed in 30 women with PMDD, while performing the point subtraction aggression paradigm. Overall, a high SPRM treatment response rate was attained (93%), in comparison with placebo (53.3%). Women with PMDD randomized to SPRM treatment had enhanced brain reactivity in the dorsal anterior cingulate cortex and dorsomedial prefrontal cortex during the aggressive response condition. The fronto-cingulate reactivity during aggressive responses depended on treatment, with a negative relationship between brain reactivity and task-related aggressiveness found in the placebo but not the SPRM group. The findings contribute to define the role of progesterone in PMDD symptomatology, suggesting a beneficial effect of progesterone receptor antagonism, and consequent anovulation, on top-down emotion regulation, i.e., greater fronto-cingulate activity in response to provocation stimuli.

## Introduction

Premenstrual dysphoric disorder (PMDD) is a mood disorder characterized by affective symptoms, such as irritability and anger, and more frequent reports of interpersonal conflicts [1, 2]. Diminished emotional regulation due to impaired impulse control is suggested to underlie negative affect in PMDD. In fact, behavioral and cognitive aspects of dysfunctional emotional regulation, like impulsivity [3], along with personality traits like neuroticism [4, 5] are related to PMDD symptom severity. Reactive aggression, defined as emotional and impulsive-directed behavior with harmful intention [6], is a diagnostic criterion of several psychiatric disorders [7, 8], and a potential outcome of irritability [9].

PMDD symptoms emerge in the early luteal phase of ovulatory cycles, when levels of estradiol and progesterone start increasing [10]. While women with PMDD do not differ from healthy women regarding their progesterone levels [11], maladaptive brain response to progesterone fluctuations is hypothesized [12], likely involving its downstream metabolite allopregnanolone [13]. Menstrual cycle-specific correlations between reactive aggression and ovarian hormone levels have been seen in naturally cycling women [14]. In mood disorders characterized by impulsiveness and emotional dysregulation, sex hormonal fluctuations potentially mediate the link between anger/irritability and lower neural inhibitory control of hormone-sensitive women, leading to reactive behavior [15, 16].

The temporal link between PMDD symptoms and progesterone fluctuations during the luteal phase, along with evidence from ovarian suppression studies, render this hormone and its downstream metabolites critical for PMDD symptomatology [12]. Indeed, ovulation suppression leads to symptom remission, whereas ovarian hormone add-back reinstates the symptoms [12]. Progesterone derivatives, such as pregnenolone and allopregnanolone, act in emotion processing brain regions as allosteric modulators of the GABA_A_ receptor [13]. Interestingly, in comparison with healthy controls, differential GABA levels have been found in women with PMDD [17, 18], while inhibition of allopregnanolone by treatment with a GABA_A_ steroid antagonist [19] or a 5α-reductase inhibitor [20] significantly reduces PMDD symptoms. Moreover, studies in rodents suggest that the anxiogenic response, which is mediated by alpha4 GABA receptor subunit upregulation following progesterone and allopregnanolone withdrawal, is another mechanism contributing to the cyclical mood symptoms [12, 21–23].

Selective progesterone receptor modulators (SPRMs) are synthetic steroids with tissue-specific activity. The second-generation of SPRM (e.g., ulipristal acetate) has antagonistic effects on the progesterone receptor [24, 25], but limited anti-glucocorticoid activity [26]. Recently, we have demonstrated that treatment with SPRM leads to a 41% reduction of affective PMDD symptoms, including marked irritability, anger and interpersonal conflicts [27]. In addition, the low-dose SPRM regimen leads to anovulation in most women, while maintaining estradiol serum concentrations at mid-follicular levels [28]. Moreover, as for GABA, progesterone receptors are present throughout the brain [29, 30], hence in place to influence the neural circuits key to cognitive and affective processing [29, 31].

Due to its emotional nature, reactive aggression can stem from provocation and frustration [6, 32]. The prevalent conceptual framework of aggression suggests altered relationships between regions involved in the top-down control (prefrontal (PFC), orbitofrontal (OFC), and anterior cingulate cortex (ACC)) and mesolimbic emotion processing regions, such as the amygdala and insula [33, 34]. The point subtraction aggression paradigm (PSAP) is a validated functional magnetic resonance imaging (fMRI) paradigm assessing reactive aggression [35]. The PSAP constitutes a monetary reward task, in which participants can either steal or protect their points after costly punishment through provocations, and targets affective misbalance and inhibitory capacity on reactive aggression and emotion processing [36]. When healthy subjects perform the PSAP, increased activation after provocation is seen in brain regions implicated in emotion, reward processing, and cognitive top-down control [37] (i.e., amygdala, striatum, medial OFC, PFC, and ACC). Notably, neuroimaging findings in PMDD indicate reduced top-down control on subcortical reactivity [38], with progesterone being associated with altered cortico-limbic reactivity during affective processing [39, 40]. However, no conclusive links have yet been established. Further, there is limited understanding of the neural substrates of female reactive aggression [41] in relation to PMDD [38].

The purpose of the present double-blinded, placebo-controlled, pharmaco-neuroimaging study was to investigate the psychoneurobiological signatures of SPRM treatment in relation to reactive aggression in women with PMDD. Specifically, the main aim was to explore whether SPRM treatment results in differential brain reactivity and functional connectivity during/in response to provocation, along with retaliatory behavior in the PSAP compared with placebo, and whether differential reactivity relates to symptom severity. Furthermore, we investigated whether treatment influenced the association between brain reactivity and task behavior, hormones, state aggression, and personality.

We hypothesized differential brain reactivity in cortico-limbic regions in response to provocation and during aggressive behavior as a result of SPRM treatment. By inducing anovulation, leading to stable and low levels of progesterone and allopregnanolone, SPRM treatment was expected to enhance cortical inhibition, either via the progesterone receptors or GABA_A_ receptors, and therefore increase frontal and decrease subcortical reactivity, along with enhancing fronto-limbic connectivity. Improved top-down control due to treatment was expected to be reflected in lower symptom severity and state aggression within the SPRM group; thus yielding less aggressive behavior. Due to lack of previous evidence on retaliatory behavior in PMDD patients, an explorative approach was undertaken. Finally, interactive effects of trait aggressiveness and neuroticism on differential brain reactivity to the PSAP were investigated.

## Materials and methods

### Participants and procedures

All procedures involving human subjects/patients were approved by the ethics committee of Uppsala (Dnr. 2016/184) and the Medical Products Agency in Sweden, EUDRA-CT 2016-001719-19. Written informed consent was obtained from all subjects; CONSORT data is presented in [27] and a flow-chart for this sub-study is presented in Supplementary Fig. S1.

The study had a multicenter, double-blind, randomized, placebo-controlled design. For this neuroimaging sub-study, we recruited 35 women with PMDD who consented to MR scanning between January 15, 2017, and October 19, 2019. All participants were from Uppsala. Of the women included in the sub-study,18 were randomized to SPRM treatment and 17 to placebo. The participants’ characteristics did not differ from those in the clinical trial [27]. Exclusion criteria were: contraindication to MRI, irregular menstrual cycle, oral contraceptive use, presence of ongoing psychiatric disorders (based on Mini-International Neuropsychiatric Interview [42]), presence of other major diseases, non-Caucasian ethnicity, and age below 20 and above 45 years.

PMDD diagnosis, according to DSM-5 criteria, was confirmed by daily symptom ratings during two consecutive menstrual cycles with the Daily Report Severity of Problems (DRSP) scale [43], using a smartphone application. The DRSP total score was generated by computing the mean of each item during the final 5 days of the premenstrual phase of the cycle and summing the 21 items. In women who had no menses during the study period, the final 5 days of the final treatment cycle were used, i.e., the approximate days of their luteal phases, had menses continued to be regular. We used the total DRSP score and the DRSP irritability, depression, affective lability, and anxiety subscales during the final treatment cycle to evaluate symptom severity (Supplementary material).

Treatment consisted of ulipristal acetate (Esmya®) 5 mg daily, or placebo, on a 3 month continuous regimen, starting on the first day of menses [27] (Supplementary material). Personality trait scores of neuroticism and aggressiveness were obtained before randomization using the Swedish universities Scale of Personality (SSP) questionnaire (Supplementary material) [44]. At baseline and the end of the final treatment cycle, all subjects filled out a battery of questionnaires on demographics, including the Aggression Questionnaire-revised Swedish version (AQ-RSV) [45].

### Hormone analyses

Venous blood samples were collected at the beginning of the MR session during the last treatment cycle to determine the levels of estradiol, progesterone, testosterone, and cortisol. Steroid hormone serum concentrations were measured at the Core Facility of Metabolomics, University of Bergen, by liquid chromatography—tandem mass spectrometry (Supplementary material).

### Task description

All participants had their brain scanned while performing the PSAP [37], during the premenstrual phase of the last treatment cycle. The paradigm consists of a computer-simulated social interaction in which participants play against a fictitious opponent, aiming to score as many points as possible, while having points stolen (i.e., provocation), being able to either steal points (aggressive response), or protect the currently owned points (i.e., protective responses). The procedure and PSAP aggression scoring are described in the Supplementary material and Fig. S2.

### MRI acquisition

Structural and functional scans were acquired on a 3.0 Tesla whole-body scanner (Achieva dStream, Philips Medical Systems, Best, The Netherlands) equipped with a 32-channel head coil. For blood oxygen dependent level (BOLD) fMRI, 240 whole-brain dynamic scans were acquired using a T2*-weighted gradient echo-planar imaging (EPI), as described in the supplementary material, with resulting images having a 1.88 × 1.88 × 2.8 mm^3^ voxel size.

### fMRI data preprocessing

fMRI data were pre-processed and analyzed using Statistical Parametric Mapping (SPM12, Wellcome Centre for Human Neuroimaging, University College London, London UK) (Supplementary material). BOLD images were spatially realigned to the first image, unwarped, corrected for slice timing, co-registered to the individual’s anatomical scan, normalized into Montreal Neurological Institute space by applying the deformation field resulting from the normalization of the anatomical image, and finally smoothed using an 8 mm FWHM Gaussian filter. Subsequently, outlier volumes were censored through the Artifact Detection Tools (http://www.nitrc.org/projects/artifact_detect) with regard to signal density (*z* > 4) and motion (movement per condition threshold >2 mm) and entered as nuisance regressors at the first level, within-subjects modelling.

### Behavioral data analyses

The change in DRSP symptom severity from before randomization to final treatment cycle was assessed by paired *t*-tests. Between-treatment comparisons for demographic data, task behavior, symptom severity, state aggression, personality, and hormonal data were performed using two-tailed two-sample *t*-tests, with Cohen’s d effect size estimate (*d*) (normally distributed data) or Mann–Whitney U test with rank-biserial (r_pb_) correlation to indicate effect size (skewed data). Bivariate and partial correlations were performed separately in each treatment group to investigate the relationships between task behavior and psychometric scores, DRSP scores, and hormonal data. In all task-related analyses we adjusted for number of provocations and total button presses. Analyses on cortisol were adjusted for the time of day when blood samples were collected. Statistical analyses were performed using the SPSS Statistics for Windows, version 26 (SPSS Inc., Chicago, Ill., USA) and significance was set at *p* ≤ 0.05.

### Neuroimaging data analyses

General linear models were used to determine condition-specific BOLD responses by regressing time series with the task conditions (Provocation (PE) > Monetary Response (MR), Aggressive Response (AR) > Monetary Response (MR), Protective Response (PR) > Monetary Response (PR), Winning Reward (WR) > Monetary Response (MR), and Stealing Reward (SR) > Monetary Response (MR)), the six head-movement parameters, and convolving with them the canonical hemodynamic response function (supplementary material). Derived single-subject contrast maps were used in second level voxel-wise analyses to examine (i) task-related effects in the whole group, (ii) treatment group differences, (iii) interaction effects between treatment and task behavior, symptom severity, state aggression, hormone levels, and personality on BOLD reactivity, and (iv) the relationship between BOLD reactivity and the aforementioned variables in each group separately. Based on the reactive aggression neural network and in line with PSAP neural correlates [37], analyses were performed using a region of interest (ROI) approach. ROIs were generated using WFU-PickAtlas as follows: the PFC including OFC as Brodmann Areas (BA) 8–14, and 44–47; ACC as BA 24, 25, and 32; striatum (i.e., putamen and caudate); bilateral insulae; and amygdalae. For voxel-wise treatment differences and interactions within the ROIs, a *p* = 0.001 uncorrected height threshold was used, combined with a *p* = 0.05 extent threshold corrected for family-wise error (FWE) [46]: *k* ≥ 111 for PFC; *k* ≥ 28 for ACC; k ≥ 22 for the insulae; *k* ≥ 25 for the striatum; and *k* ≥ 1 for the amygdalae. The effect of treatment was additionally explored within a functional ROI combining dorsal ACC (dACC) and the medial superior frontal gyrus within the dorsomedial PFC (dmPFC), based on the results of treatment differences in dACC and dmPFC anatomical ROIs. A less stringent threshold of *p* = 0.001, uncorrected, with a minimum cluster size of ten voxels, was used to test correlations in each treatment group separately. Statistical procedures are described in the supplementary material. Mean signal time-course was extracted and plotted for the differentially activated ROIs.

### Psychophysiological interaction (PPI) analyses

In order to investigate potential treatment effects on functional connectivity during AR, we employed psychophysiological interaction (PPI) analysis using the SPM12 software package [47, 48], with the dmPFC and dACC clusters that differed between groups in the contrast AR > MR as seed regions. Thus, the functional coupling between these ROIs and distant brain voxels was assessed. Estimation of single-subject matrices for PPI effects were similar to the ones used to investigate main task effects, but included the seed region’s mean signal time-course and PPI as regressors. BOLD signal time series were extracted from these ROIs using the VOI time series as physiological regressors and the main effect of condition (AR > MR) as the psychological regressor. To explore whether activity in other brain regions correlated with the dACC or mPFC for the AR condition (AR > MR), the individual contrast images were then taken onto group-level analysis. As no significant functional connectivity was detected on the whole group level between neither of the two seed regions and distant voxels, differential functional connectivity patterns between the groups could not be assessed.

## Results

### Demographic, clinical and behavioral characteristics

**Fig. 1:**
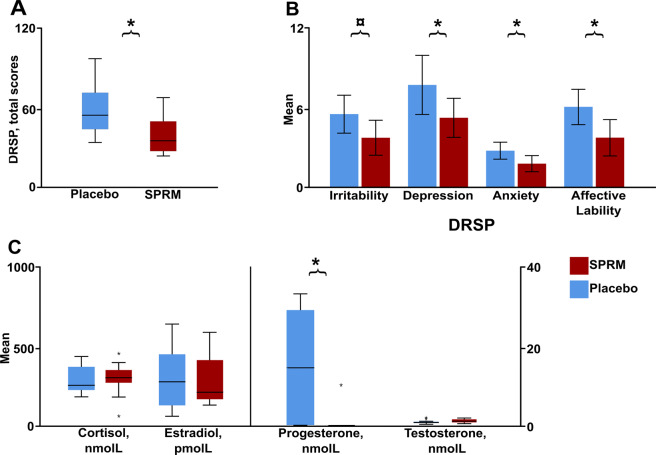
Differences between groups in symptom severity and hormonal levels. Descriptive panel showing the effect of SPRM treatment in comparison with placebo, according to the Daily Record of Severity of Problems (DRSP) total score (*d* = 1.09) (**A**), the four subscales, depression, anxiety, affective lability, and irritability (**B**), and hormone levels, indicated by the mean concentrations of estradiol, progesterone, and testosterone (**C**). Error bars indicate 95% confidence intervals. *: *p* ≤ 0.05; ¤: *p* ≤ 0.1.

Demographics, task behavior, symptom severity, state aggression, and hormonal data of the participants are presented in Table 1. Four women (three receiving SPRM treatment, one placebo) were excluded from the analyses because they used the AR button less than three times. In addition, one woman in the placebo group was excluded due to excessive movement artifacts. Thus, 30 women with PMDD were available for analyses (SPRM: *n* = 15, Table 1). Before randomization, the two treatment groups did not differ in total DRSP symptom severity (total sample, *M* = 68.6 ± 15.7), AQ-RSV (total sample *M* = 38.8 ± 12.3) or in personality scores of aggressiveness and neuroticism (Table 1). Progesterone levels were substantially lower in the SPRM group in the final treatment cycle, clearly outside luteal phase levels (Fig. 1), while cortisol, estradiol and testosterone levels did not differ (Table 1).

**Table 1:** Demographics, DRSP, psychometrics, task behavior and hormone levels in women with PMDD, randomized to SPRM or placebo.

	Placebo	SPRM
	(*n* = 15)	(*n* = 15)
Age, years^a^	35 ± 8	34 ± 5
Age at PMDD onset, years	23 ± 6	25 ± 6
Educational level		
University degree	11 (73)	11 (73)
No university degree	4 (27)	4 (27)
Employment		
Working part- or full time	12 (80)	14 (93)
Studying	2 (13)	1 (7)
Other	1 (7)	0 (0)
Personality factors		
SSP Neuroticism	324 ± 49	314 ± 56
SSP Aggressiveness	154 ± 19	144 ± 23
Mood at baseline		
Total DRSP score	69 ± 12	68 ± 19
AQ-RSV Score	43 ± 10	36 ± 13
Mood during treatment		
Total DRSP score	58 ± 20	38 ± 16*
DRSP subscale scores		
Irritability^a^	6 ± 2	4 ± 2^¤^
Depression	8 ± 4	5 ± 2*^d1^
Anxiety^a^	3 ± 1	2 ± 1*^rpb1^
Affective lability^a^	6 ± 2	4 ± 2*^rpb2^
AQ-RSV Score	40 ± 14	28 ± 13*^d2^
PSAP behavioral outcomes		
PSAP aggression score^a^	9 ± 6	10 ± 9
Earning presses, n^a^	1776 ± 407	1683 ± 570
Aggressive responses, n	273 ± 164	279 ± 179
Protective responses, n^a^	255 ± 131	169 ± 154*^rpb3^
Total Button Presses, n^a^	2304 ± 285	2130 ± 405
Points Earned, n	3 ± 4	1 ± 5
Provocations, n^a^	14 ± 2	16 ± 3
Option 2 over Provocations, n	20 ± 13	19 ± 14
Option 3 over Provocations, n^a^	18 ± 10	12 ± 11^¤ rpb4^
Hormonal levels during treatment		
Estradiol, pmol/L	350 ± 207	322 ± 211
Progesterone, nmol/L^a^	14.4 ± 13.7	1.7 ± 3.7*^rpb5^
Testosterone, nmol/L	0.8 ± 0.4	1 ± 0.3
Cortisol, nmol/L	251 ± 87	270 ± 98

Data presented as mean ± SD or n (%).

*AQ-RSV* Aggression Questionnaire-revised Swedish version, *DRSP* Daily Record of Severity of Problems, *PMS* Premenstrual Syndrome, *PSAP* Point Substraction Aggression Paradigm.

^*^: *p* ≤ 0.05; ¤: *p* ≤ 0.15.

^a^Deviated from normality, Mann–Whitney U test was performed.

^d1^Cohen’s d effect size: *d* = 0.77.

^d2^Cohen’s d effect size: *d* = 0.96.

^rpb1^Rank-biserial correlation effect size: r_pb_ = 0.54.

^rpb2^Rank-biserial correlation effect size: r_pb_ = 0.58.

^rpb3^Rank-biserial correlation effect size: r_pb_ = 0.42.

^rpb4^Rank-biserial correlation effect size: rp_b_ = 0.4.

^rpb5^Rank-biserial correlation effect size: r_pb_ = 0.64.

The SPRM group reported lower scores both in the total DRSP and the subscales during treatment, compared with placebo, although irritability was only borderline significant (*p* = 0.055) (Fig. 1). Treatment response to SPRM was higher (93.3%) compared with placebo (53.3%) (*χ*^*2*^ = 5.18, *df* = 1, *p* = 0.02). Women randomized to SPRM displayed lower aggression during treatment as measured by AQ-RSV (Table 1).

As to the PSAP task behavior; women randomized to SPRM used PRs less often than women on placebo; otherwise no behavioral differences were noted between treatment groups (Table 1). The number of aggressive button presses and the PSAP aggression score were positively correlated with irritability scores in the SPRM group (*r*_*Pearson*_ (7) = 0.69, *p* = 0.025 and *r*_*Pearson*_ (7) = 0.71, *p* = 0.02, respectively). We noted no relationship between AQ-RSV or personality scores and task behavior in any treatment group. Hormonal levels did not correlate with PSAP behavior.

### Functional neuroimaging results

#### Task-related BOLD reactivity

The main task effects are presented in Table S1. During the provocation condition (contrast PE > MR) reactivity was found in the right insula and in three clusters in the PFC (the right precentral gyrus, the right supplementary motor area, and in the left precentral gyrus). In addition, we noted significant BOLD reactivity in the left amygdala and in the left caudate nucleus during provocation.

During the winning condition (contrast MR > WR), one cluster was significantly deactivated in the orbital part of the right medial frontal gyrus. Otherwise, no significant BOLD reactivity was observed in any of the ROIs for any of the other conditions (AR > ΜR, SR > ΜR, PR > ΜR). Task effects by treatment group are presented in Table S2.

#### Treatment-related BOLD reactivity

**Fig. 2:**
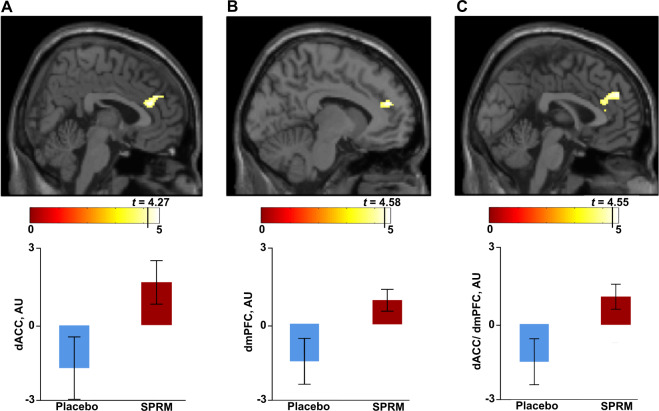
Increased fronto-cingulate reactivity in women receiving SPRM compared with placebo during aggressive response. SPRM treatment in women with PMDD (*n* = 15) led to enhanced BOLD reactivity (SE: 95% CI) in the contrast aggressive response > monetary response, when compared to the placebo (*n* = 15), for the following clusters within two regions of interest: (**A**) the dACC (two maxima: [x, y, z: 4, 34, 20 and 4, 40, 28]; *k* = 106; *T* = 4.27; *p*_*FWE-cluster*_ = 0.005), (**B**) the dmPFC (two maxima: [x, y, z: 0, 46, 30 and −8, 46, 20]; *k* = 141; *T* = 4.58; *p*_*FWE-cluster*_ = 0.029), and (**C**) within the combined dACC/ dmPFC ROI mask (three maxima: [x, y, z: 0, 46, 30, −8, 46, 20, and 4, 34, 20]; *k* = 199; *T* = 4.55; *p*_*FWE-cluster*_ = 1.76E-05_)_. The first maximum is illustrated in the graph. The mean time-course of the signal was extracted to visualize the treatment group differences in BOLD response.

We next compared the PSAP-induced BOLD signal between SPRM treatment and placebo. Aggressive response (AR) was associated with greater BOLD signal in the dACC and in the dmPFC (i.e., BA 9 and 24/32) among women randomized to SPRM (Fig. 2A and B). No additional difference in BOLD reactivity was observed in other ROIs during AR. Further, we found no treatment-dependent differences in BOLD reactivity during the provocation, winning and SR, and PR conditions.

Since reactivity in the dACC and dmPFC was highly correlated (*r*_*Pearson*_*(30)* = 0.874; *p* < 0.001), the groups were compared using a mask including the differentially activated clusters in these two regions. Enhanced reactivity in a bilateral cluster extending over both dACC and dmPFC was found in women randomized to SPRM, suggesting both regions are implicated in the same process (Fig. 2C).

**Fig. 3:**
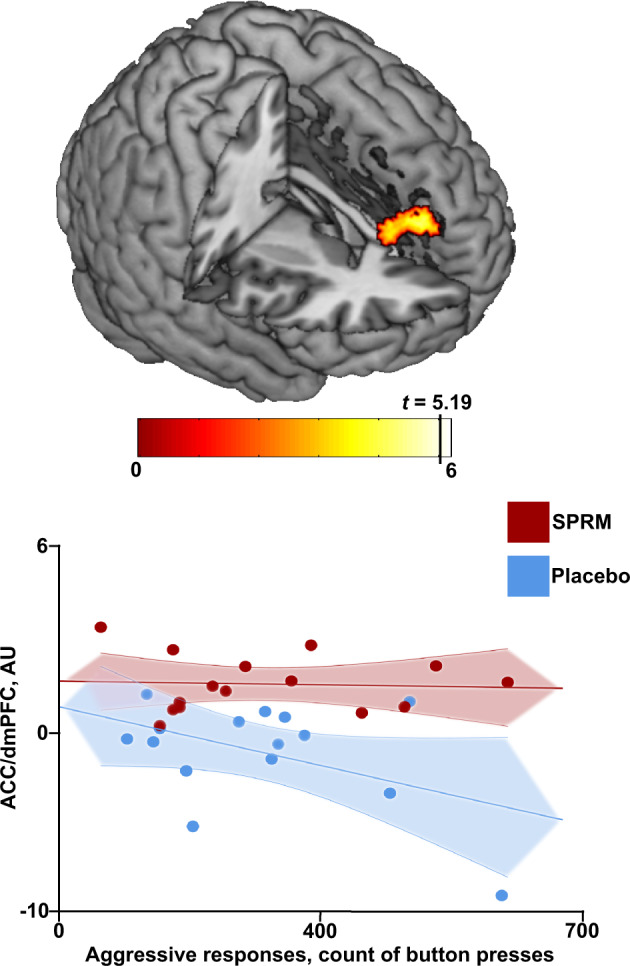
Task behavior (aggressive responses) by treatment interaction on dACC/ dmPFC (two maxima: [x, y, z: 0, 46, 30 and 4, 34, 20], *k* = 144, *T* = 5.19, *p*_*FWE-cluster*_ = 1.76E-05) BOLD response for the T-contrast Aggressive response > Monetary response (color scaled by interaction *t* statistic). The first maximum is illustrated in the graph. The parameter estimates for the peak maxima of the significant interaction clusters were extracted for visualization purposes.

Regarding task behavior, a treatment-by-ARs interaction was found in the dACC/dmPFC, depicted as a negative correlation between the fronto-cingulate BOLD reactivity and ARs in the placebo group only (Fig. 3). The post-hoc analyses revealed a negative correlation between BOLD and ARs in the placebo group on a trend level (*p* < 0.005 uncorrected). No significant interaction effects were noted between treatment groups and symptom severity, state aggression, personality, or hormones on brain reactivity.

#### Functional connectivity (PPI) analyses

Aggressive behavior-dependent neural reactivity in the seed regions, dACC and dmPFC, did not functionally correlate with reactivity in other brain regions in the whole sample, and thus no group differences in functional connectivity could be assessed.

## Discussion

SPRM treatment was associated with greater fronto-cingulate reactivity, specifically in the dACC and dmPFC during ARs to provocation in women with PMDD. Considering that aggression is a behavioral outcome of irritability [49, 50], and that SPRM treatment is associated with lower irritability symptoms [27], this finding offers a plausible mechanism by which progesterone receptor antagonism, and consequent anovulation, facilitates emotion regulation in PMDD.

The present findings represent the first pharmaco-neuroimaging indication of progesterone as modulator of fronto-cingulate circuits behind reactive aggression in PMDD. The ACC and mPFC regions are involved in aggression, cognitive control, and negative affect in healthy individuals [41]. They have been identified as nodes of frontal attentional and control networks, and shown to be influenced by hormonal fluctuations during the menstrual cycle phase, and by oral contraceptive use, in healthy women [38, 51]. Lower cortical inhibition in frontal regions may indeed precipitate PMDD symptoms such as irritability [17, 52], and in line with this, women with PMDD display blunted fronto-cingulate reactivity to emotional stimuli in comparison with controls [53], likely as a sign of poor top-down control on emotions.

As part of the dorsal top-down control system, both the dACC and dmPFC are implicated in conscious emotional reappraisal following negative stimuli presentation [54]. In PMDD, the role of progesterone (or allopregnanolone) in emotional regulation is presumably exerted through its influence on amygdala-ACC coupling [51]. Lower luteal phase ACC reactivity to social negative stimuli has been observed in women with PMDD [39]. The dACC has further been implicated in experimental settings that require conflict resolving, where its central role is cognitive control adjustment [55, 56]. Reactivity of the dACC has also been observed in the decision phase of the Taylor Aggression Paradigm [55]. The current findings are thus in line with the region’s emotion-cognition conflict processing role [57], as differential reactivity occurred when the participants were asked to decide between two alternatives (retaliation vs. protection of already acquired points). This is the first evidence of dACC reactivity following ARs in PMDD. Paradigms distinguishing between low and high provocation conditions [55] would further inform on the emotional and conflict resolution processes in PMDD.

Moreover, the dorsolateral PFC (dlPFC) is an inhibitory control region and its reactivity has been linked to PMDD [52], and proved to be sensitive to progesterone and estradiol fluctuations [58]. During anticipation of negative emotional stimuli, dlPFC and mPFC show heightened luteal reactivity in women with PMDD [40]. This is the first study to link progesterone to the dmPFC and emotional processing, suggesting improved inhibitory top-down control for the patients receiving SPRM treatment. Moreover, as region-specific influences of treatment could not be distinguished, the same functional effect during aggressive responding could be attributed to both regions.

Regarding behavior, as expected, the higher the irritability, the more aggressively the participants behaved during the task. Greater ARs were correlated on a trend level (likely due to the sample size) with lower fronto-cingulate reactivity in the placebo group only, further supporting an effect of SPRM treatment on control over reactive aggression. Nevertheless, we found no behavioral differences between the treatment groups. This finding can be attributed to either low sensitivity of the behavioral measures in capturing treatment effects or that the women included in this study were not a highly aggressive sample. The scores in the current sample at baseline and for the SPRM group during treatment (the placebo group had marginally higher mean, see Table 1) were lower than the cut-off score for aggressive behavior [45], as well as than those observed in general (females: 52) and clinical populations (females with persistent depressive disorder: 59) [59]. SPRM treatment was associated with lower state aggression compared to placebo, although the scores did not correlate with brain reactivity. It is plausible that there is more to the relationship between irritability and aggression. Aggression is only one of the possible outcomes of irritability [9] and needs to be addressed by a multifaceted evaluation of impulsive/aggressive behavior, along with a larger sample. Assessment of task adherence could also inform whether the observed response is generated by reactive aggression. Furthermore, the relationship between symptom severity and fronto-cingulate reactivity did not vary by treatment group, despite SPRM treatment leading to lower irritability [27], which also suggests a somewhat complex relationship between progesterone, irritability, and aggression. Even though neuroticism constitutes a candidate factor in predicting luteal phase negative affect [4], no predisposition emerged when considering trait neuroticism and trait aggressiveness, or their relationship to treatment-specific brain reactivity. Last, because only one subject in the SPRM treatment group was categorized as non-remitter, the relationship between treatment efficacy and brain reactivity could not be assessed, thus impeding prediction analyses.

Emotion regulation can be characterized as the, conscious or not, subjective perception and evaluation of external events [60], requiring global network efficiency [61]. In our sample of women with PMDD, the task generated prefrontal and subcortical reactivity, along with orbital medial frontal gyrus deactivation during the provocation and WR conditions; which is in line with the PSAP-reactivity patterns observed in healthy [37] and aggressive individuals [33], respectively. In fMRI adaptations of aggression paradigms, reactive aggression is considered a product of a distributed cortico-limbic network responsible for emotional regulation in healthy men [62] and, accordingly, heightened amygdala and striatal activation, accompanied by lower functional connectivity with prefrontal regions, was noted in violent subjects [63]. SPRM treatment did not influence other subcortical regions or the OFC, neither functional connectivity. The lack of significant reactivity in subcortical regions in response to the AR condition, may explain why we did not observe any connectivity patterns. This could indicate low sensitivity of the task in detecting brain reactivity in a moderately aggressive sample, combined with the modest fMRI spatial resolution. The use of a baseline condition, entailing no or minimal cognitive activity, such as a fixation cross could have potentially increased the sensitivity of the task. Nevertheless, replication of these results in a larger sample is warranted.

Concerning the neuroendocrine underpinnings, SPRM treatment leads to anovulation and low levels of progesterone and likely allopregnanolone. In addition, as SPRM treatment maintains estradiol on mid-follicular levels, it cannot be excluded that the present findings were merely an outcome of progesterone receptor antagonism [27]. In fact, following ovarian suppression, PMDD symptom reinstatement has been noticed after adding-back of either estradiol or progesterone [64] or their combination [65], but the distinct effects of the two hormones have not yet been discerned. The present study does not allow to assess whether progesterone alone, or together with its metabolites, mediates the treatment effect. It can be hypothesized that lower cortical inhibition during the symptomatic phase precipitates PMDD symptoms such as irritability [17], as an outcome of altered GABA_A_ receptor sensitivity to allopregnanolone in emotionally relevant ROIs, including the ACC and mPFC [18]. Further studies are thus needed to disentangle the effects of progesterone and allopregnanolone, and even estradiol, in mediating the treatment effect on the neural correlates of PMDD.

As to the usefulness of SPRMs in women with PMDD, further studies are clearly needed. Despite isolated cases of severe adverse effects on liver function in older subjects treated for uterine fibroids with SPRM [66], the 3 month treatment regimen (even up to four three-month courses) has been linked to stable or decreasing side effects [27, 67]. This together with its positive effects on depressive and irritability symptoms, and with the ongoing development of SPRMs with safer profiles [68], highlights the relevance of pharmaco-neuroimaging studies targeting potential mechanisms behind PMDD.

In conclusion, SPRM treatment, which reduced PMDD symptom severity [27], was associated with greater top-down control during aggressive behavior. The present results indeed highlight optimistic prospects for treatment based on SPRM to target brain circuits of clinical and behavioral relevance. Here, a randomized placebo controlled design and analyses accounting for multiple comparisons, provide evidence on treatment differences, which points to the influence of progesterone (or downstream allopregnanolone) on fronto-cingulate functioning in women with PMDD [66, 68].

## Funding and disclosure

This study was supported by the Swedish Research Council (2016-01439, 2020-01801), the Swedish Society of Medicine (SLS-573171, SLS-597211, SLS-789101) and the Swedish Brain Foundation (2020-0255). EC is a Marie Skłodowska Curie fellow and receives funds from the Swedish Research Council (2015-00495), EU FP7-People-Cofund (INCA 600398) and SciLifeLab. Gedeon Richter provided study drugs, but no other financial support.

VGF declares that she has received a honorarium as a consultant for SAGE Therapeutics. RL received travel grants and/or conference speaker honoraria within the last 3 years from Bruker BioSpin MR, Heel, and support from Siemens Healthcare regarding clinical research using PET/MR. He is a shareholder of the start-up company BM Health GmbH since 2019. ISP has served occasionally on advisory boards or acted as invited speaker at scientific meetings for Asarina Pharma, Bayer HealthCare, Gedeon Richter, Peptonics, Shire/Takeda, Sandoz, and Lundbeck A/S. All other authors declare that they have no competing interests.

## Supplementary information

SI

## References

[1] Rapkin AJ, Winer SA (2009). Premenstrual syndrome and premenstrual dysphoric disorder: quality of life and burden of illness. Expert Rev Pharmacoecon Outcomes Res..

[2] Ko C-H, Long C-Y, Chen S-YCI-J, Huang T-H, Yen J-Y (2013). Depression, Irritability, and Anxiety in Women with Premenstrual Dysphoric Disorder. Int J Psychiatry Med.

[3] Dawson DN, Eisenlohr-Moul TA, Paulson JL, Peters JR, Rubinow DR, Girdler SS (2018). Emotion-related impulsivity and rumination predict the perimenstrual severity and trajectory of symptoms in women with a menstrually related mood disorder. J Clin Psychol..

[4] Gingnell M, Comasco E, Oreland L, Fredrikson M, Sundström-Poromaa I (2010). Neuroticism-related personality traits are related to symptom severity in patients with premenstrual dysphoric disorder and to the serotonin transporter gene-linked polymorphism 5-HTTPLPR. Arch Women’s Ment Health.

[5] Adewuya AO, Loto OM, Adewumi TA (2009). Pattern and correlates of premenstrual symptomatology amongst Nigerian University students. J Psychosom Obstet Gynecol.

[6] Anderson CA, Bushman BJ. Human aggression. Annu Rev Psychol. 2002;53:27–51. 10.1146/annurev.psych.53.100901.135231 11752478

[7] McCloskey MS, New AS, Siever LJ, Goodman M, Koenigsberg HW, Flory JD (2009). Evaluation of behavioral impulsivity and aggression tasks as endophenotypes for borderline personality disorder. J Psychiatr Res.

[8] Coccaro EF (2012). Intermittent Explosive Disorder as a Disorder of Impulsive Aggression for DSM-5. Am J Psychiatry.

[9] Yager J (2020). Irritability Disorders in Adults: Diagnostic Categories Missing in Plain Sight?. J Nerv Ment Dis.

[10] Epperson CN, Steiner M, Hartlage SA, Eriksson E, Schmidt PJ, Jones I (2012). Premenstrual dysphoric disorder: evidence for a new category for DSM-5. Am J Psychiatry.

[11] Bäckström T, Andreen L, Birzniece V, Björn I, Johansson I-M, Nordenstam-Haghjo M (2003). The role of hormones and hormonal treatments in premenstrual syndrome. CNS Drugs.

[12] Sundström-Poromaa I, Comasco E, Sumner R, Luders E (2020). Progesterone - Friend or foe?. Front Neuroendocrinol.

[13] Bäckström T, Bixo M, Johansson M, Nyberg S, Ossewaarde L, Ragagnin G (2014). Allopregnanolone and mood disorders. Prog Neurobiol.

[14] Geniole SN, MacDonell ET, McCormick CM (2017). The Point Subtraction Aggression Paradigm as a laboratory tool for investigating the neuroendocrinology of aggression and competition. Hormones Behav.

[15] Peters JR, Owens SA, Schmalenberger KM, Eisenlohr-Moul TA (2020). Differential effects of the menstrual cycle on reactive and proactive aggression in borderline personality disorder. Aggress Behav..

[16] Petersen N, London ED, Liang L, Ghahremani DG, Gerards R, Goldman L (2016). Emotion regulation in women with premenstrual dysphoric disorder. Arch Women’s Ment Health.

[17] Epperson CN, Haga K, Mason GF, Sellers E, Gueorguieva R, Zhang W (2002). Cortical γ-aminobutyric acid levels across the menstrual cycle in healthy women and those with premenstrual dysphoric disorder: a proton magnetic resonance spectroscopy study. Arch Gen Psychiatry.

[18] Liu B, Wang G, Gao D, Gao F, Zhao B, Qiao M (2015). Alterations of GABA and glutamate–glutamine levels in premenstrual dysphoric disorder: a 3T proton magnetic resonance spectroscopy study. Psychiatry Res: Neuroimaging.

[19] Bixo M, Ekberg K, Poromaa IS, Hirschberg AL, Jonasson AF, Andréen L (2017). Treatment of premenstrual dysphoric disorder with the GABAA receptor modulating steroid antagonist Sepranolone (UC1010)—A randomized controlled trial. Psychoneuroendocrinology..

[20] Martinez PE, Rubinow DR, Nieman LK, Koziol DE, Morrow AL, Schiller CE (2016). 5α-Reductase Inhibition Prevents the Luteal Phase Increase in Plasma Allopregnanolone Levels and Mitigates Symptoms in Women with Premenstrual Dysphoric Disorder. Neuropsychopharmacology..

[21] Smith SS, Gong QH, Li X, Moran MH, Bitran D, Frye CA (1998). Withdrawal from 3alpha-OH-5alpha-pregnan-20-One using a pseudopregnancy model alters the kinetics of hippocampal GABAA-gated current and increases the GABAA receptor alpha4 subunit in association with increased anxiety. J Neurosci..

[22] Smith SS, Gong QH, Hsu F-C, Markowitz RS, Li X (1998). GABA A receptor α4 subunit suppression prevents withdrawal properties of an endogenous steroid. Nature..

[23] Gulinello M, Gong QH, Li X, Smith SS (2001). Short-term exposure to a neuroactive steroid increases α4 GABAA receptor subunit levels in association with increased anxiety in the female rat. Brain Res.

[24] Nallasamy S, Kim J, Sitruk-Ware R, Bagchi M, Bagchi I (2013). Ulipristal Blocks Ovulation by Inhibiting Progesterone Receptor—Dependent Pathways Intrinsic to the Ovary. Reprod Sci.

[25] Esber N, Le Billan F, Resche-Rigon M, Loosfelt H, Lombès M, Chabbert-Buffet N (2015). Ulipristal Acetate Inhibits Progesterone Receptor Isoform A-Mediated Human Breast Cancer Proliferation and BCl2-L1 Expression. PLOS ONE.

[26] Rosato E, Farris M, Bastianelli C (2016). Mechanism of Action of Ulipristal Acetate for Emergency Contraception: a Systematic Review. Front Pharm.

[27] Comasco E, Kallner HK, Bixo, M, Hirschberg, AL, Nyback, S, de Grauw, H, et al. Ulipristal acetate for treatment of premenstrual dysphoric disorder – a proof-of-concept randomized controlled trial. Am J Psychiatry. 2021;178:256–65. 10.1176/appi.ajp.2020.20030286 33297719

[28] Whitaker LH, Williams AR, Critchley HO (2014). Selective progesterone receptor modulators. Curr Opin Obstet Gynecol.

[29] Brinton RD, Thompson RF, Foy MR, Baudry M, Wang J, Finch CE (2008). Progesterone receptors: form and function in brain. Front Neuroendocrinol.

[30] Barth C, Villringer A, Sacher J. Sex hormones affect neurotransmitters and shape the adult female brain during hormonal transition periods. Front Neurosci. 2015;9:37. 10.3389/fnins.2015.00037 PMC4335177 25750611

[31] Witte AV, Savli M, Holik A, Kasper S, Lanzenberger R (2010). Regional sex differences in grey matter volume are associated with sex hormones in the young adult human brain. NeuroImage..

[32] Berkowitz L. Aggression: Its causes, consequences, and control. Mcgraw-Hill Book Company; New York, NY, England; 1993.

[33] Coccaro EF, McCloskey MS, Fitzgerald DA, Phan KL (2007). Amygdala and orbitofrontal reactivity to social threat in individuals with impulsive aggression. Biol Psychiatry.

[34] Gan G, Preston-Campbell RN, Moeller SJ, Steinberg JL, Lane SD, Maloney T (2016). Reward vs. retaliation—The role of the mesocorticolimbic salience network in human reactive aggression. Front Behav Neurosci.

[35] Kose S, Steinberg JL, Moeller FG, Gowin JL, Zuniga E, Kamdar ZN (2015). Neural correlates of impulsive aggressive behavior in subjects with a history of alcohol dependence. Behav Neurosci.

[36] Siever LJ (2008). Neurobiology of aggression and violence. Am J Psychiatry.

[37] Skibsted AP, Cunha‐Bang SD, Carré JM, Hansen AE, Beliveau V, Knudsen GM (2017). Aggression‐related brain function assessed with the Point Subtraction Aggression Paradigm in fMRI. Aggressive Behav.

[38] Dubol M, Epperson CN, Lanzenberger R, Sundström-Poromaa I, Comasco E. Neuroimaging premenstrual dysphoric disorder: a systematic and critical review. Front Neuroendocrinol. 2020;57:100838. 10.1016/j.yfrne.2020.100838 32268180

[39] Gingnell M, Ahlstedt V, Bannbers E, Wikström J, Sundström-Poromaa I, Fredrikson M (2014). Social stimulation and corticolimbic reactivity in premenstrual dysphoric disorder: a preliminary study. Biol Mood Anxiety Disord.

[40] Gingnell M, Bannbers E, Wikström J, Fredrikson M, Sundström-Poromaa I (2013). Premenstrual dysphoric disorder and prefrontal reactivity during anticipation of emotional stimuli. Eur Neuropsychopharmacol.

[41] Denson TF, O’Dean SM, Blake KR, Beames JR (2018). Aggression in women: behavior, brain and hormones. Front Behav Neurosci.

[42] Sheehan DV, Lecrubier Y, Sheehan KH, Amorim P, Janavs J, Weiller E (1998). The Mini-International Neuropsychiatric Interview (M.I.N.I.): the development and validation of a structured diagnostic psychiatric interview for DSM-IV and ICD-10. J Clin Psychiatry.

[43] Endicott J, Nee J, Harrison W (2006). Daily Record of Severity of Problems (DRSP): reliability and validity. Arch Women’s Ment Health.

[44] Gustavsson JP, Bergman H, Edman G, Ekselius L, Von Knorring L, Linder J (2000). Swedish universities Scales of Personality (SSP): construction, internal consistency and normative data. Acta Psychiatr Scandinavica.

[45] Prochazka H, Agren H (2001). Aggression in the general Swedish population, measured with a new self-rating inventory: the Aggression Questionnaire-revised Swedish version (AQ-RSV). Nord J Psychiatry.

[46] Eklund A, Nichols TE, Knutsson H (2016). Cluster failure: why fMRI inferences for spatial extent have inflated false-positive rates. Proc Natl Acad Sci.

[47] Friston K, Buechel C, Fink G, Morris J, Rolls E, Dolan RJ (1997). Psychophysiological and modulatory interactions in neuroimaging. Neuroimage..

[48] Gitelman DR, Penny WD, Ashburner J, Friston KJ (2003). Modeling regional and psychophysiologic interactions in fMRI: the importance of hemodynamic deconvolution. NeuroImage..

[49] Leibenluft E (2017). Pediatric Irritability: a Systems Neuroscience Approach. Trends Cogn Sci.

[50] Caprara GV, Cinanni V, D’imperio G, Passerini S, Renzi P, Travaglia G (1985). Indicators of impulsive aggression: present status of research on irritability and emotional susceptibility scales. Personal Individ Differ.

[51] Toffoletto S, Lanzenberger R, Gingnell M, Sundström-Poromaa I, Comasco E (2014). Emotional and cognitive functional imaging of estrogen and progesterone effects in the female human brain: a systematic review. Psychoneuroendocrinology..

[52] Schiller CE, Johnson SL, Abate AC, Schmidt PJ, Rubinow DR (2011). Reproductive steroid regulation of mood and behavior. Compr Physiol.

[53] Comasco E, Hahn A, Ganger S, Gingnell M, Bannbers E, Oreland L (2014). Emotional fronto-cingulate cortex activation and brain derived neurotrophic factor polymorphism in premenstrual dysphoric disorder. Hum Brain Mapp.

[54] Etkin A, Egner T, Kalisch R (2011). Emotional processing in anterior cingulate and medial prefrontal cortex. Trends Cogn Sci..

[55] Krämer UM, Jansma H, Tempelmann C, Münte TF (2007). Tit-for-tat: the neural basis of reactive aggression. Neuroimage..

[56] Botvinick MM, Cohen JD, Carter CS (2004). Conflict monitoring and anterior cingulate cortex: an update. Trends Cogn Sci.

[57] Etkin A, Egner T, Peraza DM, Kandel ER, Hirsch J (2006). Resolving emotional conflict: a role for the rostral anterior cingulate cortex in modulating activity in the amygdala. Neuron..

[58] Amin Z, Epperson CN, Constable RT, Canli T (2006). Effects of estrogen variation on neural correlates of emotional response inhibition. NeuroImage..

[59] Prochazka H, Agren H (2003). Self-rated aggression and cerebral monoaminergic turnover. Sex differences in patients with persistent depressive disorder. Eur Arch Psychiatry Clin Neurosci..

[60] Etkin A, Büchel C, Gross JJ (2015). The neural bases of emotion regulation. Nat Rev Neurosci.

[61] Pan J, Zhan L, Hu C, Yang J, Wang C, Gu L (2018). Emotion Regulation and Complex Brain Networks: association Between Expressive Suppression and Efficiency in the Fronto-Parietal Network and Default-Mode Network. Front Hum Neurosci.

[62] Repple J, Pawliczek CM, Voss B, Siegel S, Schneider F, Kohn N (2017). From provocation to aggression: the neural network. BMC Neurosci.

[63] da Cunha-Bang S, Fisher PM, Hjordt LV, Perfalk E, Persson Skibsted A, Bock C, et al. Violent offenders respond to provocations with high amygdala and striatal reactivity. Social Cognitive and Affective Neuroscience. 2017;12:802-10. 10.1093/scan/nsx006 PMC5460055 28338916

[64] Schmidt PJ, Nieman LK, Danaceau MA, Adams LF, Rubinow DR (1998). Differential behavioral effects of gonadal steroids in women with and in those without premenstrual syndrome. N Engl J Med.

[65] Schmidt PJ, Martinez PE, Nieman LK, Koziol DE, Thompson KD, Schenkel L (2017). Premenstrual dysphoric disorder symptoms following ovarian suppression: triggered by change in ovarian steroid levels but not continuous stable levels. Am J Psychiatry.

[66] Middelkoop M-A, Huirne JAF, van der Weide MCJ, Bosmans JE, Hehenkamp WJK (2021). A multi-centre, randomized, non-inferiority trial to compare ulipristal with standard surgical treatment in women with symptomatic uterine fibroids: protocol of the MYOMEX-2 trial. Eur J Obstet Gynecol Reprod Biol.

[67] Rabe T, Saenger N, Ebert AD, Roemer T, Tinneberg H-R, De Wilde RL (2018). Selective Progesterone Receptor Modulators for the Medical Treatment of Uterine Fibroids with a Focus on Ulipristal Acetate. BioMed Res Int.

[68] Möller C, Bone W, Cleve A, Klar U, Rotgeri A, Rottmann A (2018). Discovery of vilaprisan (BAY 1002670): a highly potent and selective progesterone receptor modulator optimized for gynecologic therapies. ChemMedChem..

