# Supplementary material for: Brain reactivity during aggressive response in women with premenstrual dysphoric disorder treated with a selective progesterone receptor modulator
Source: Neuropsychopharmacology. 2021 Apr 29;46(8):1460–7. doi: 10.1038/s41386-021-01010-9 (PMC8209206; doi:10.1038/s41386-021-01010-9)
Supplement: Supplementary file 1 — SI [file 41386_2021_1010_MOESM1_ESM.docx]

Supplementary Information

Brain reactivity during aggressive response in patients with premenstrual dysphoric disorder treated with selective progesterone receptor modulator

Elisavet Kaltsouni^1^, MSc, Patrick M. Fisher^2^, PhD, Manon Dubol^1^, PhD, Steinar Hustad^3^, PhD, Rupert Lanzenberger^4^, MD, PD, Vibe G. Frokjaer^2,5^, MD, PhD, Johan Wikström^6^, MD, PhD, Erika Comasco^1^*, PhD, Inger Sundström-Poromaa^7^*, MD, PhD

Supplementary methods and materials

The DRSP scale. The DRSP instrument constitutes a daily rating list of PMDD physical and psychological symptoms through 21 items scored on a 6-point severity scale, in the different phases of the menstrual cycle in order to confirm the diagnosis. The DRSP subscales were computed using the corresponding DRSP items related to the four core symptoms for PMDD diagnosis according to DSM-5 criteria, as described in [1,2]. Items were grouped into an anger/irritability subscale (ie, anger and/or irritability and conflicts with people), a depressive symptoms subscale (ie, felt depressed, felt hopeless, felt worthless or guilty), an affective lability subscale (ie, had mood swings, was more easily hurt), and an anxiety subscale (ie, felt more anxious) [1].

Clinical trial and treatment. The low-dose continuous SPRM treatment induces anovulation in most women [3], which together with progesterone receptor antagonism, are the targets for the expected symptom relief in PMDD patients. Moreover, ulipristal acetate is associated with a very beneficial side-effect profile, which is no different from placebo [4,5]. Ulipristal acetate (Esmya®) and identical-looking placebo tablets were administered to the women, after randomization, starting on the first day of menses[5]. The functional MRI scan took place at the end of the third, and final, treatment cycle, which is hereafter referred to as scan month. In women who maintained regular menstrual cycles during the study, the final scan was scheduled in the luteal phase of the menstrual cycle. Treatment response was evaluated as described in[5].

Personality assessment. The Swedish university Scales of Personality (SSP) [6] is a self-rating scale comprising 91 items grouped in 13 subscales (Somatic Trait Anxiety, Psychic Trait Anxiety, Stress Susceptibility, Lack of Assertiveness, Impulsiveness, Adventure Seeking, Detachment, Social Desirability, Embitterment, Trait Irritability, Mistrust, Verbal Trait Aggression, Physical Trait Aggression), which form 3 main factors: SSP Neuroticism (Somatic Trait Anxiety, Psychic Trait Anxiety, Stress Susceptibility, Lack of Assertiveness, Embitterment, Mistrust), Aggressiveness (Trait Irritability, Verbal Trait Aggression, Physical Trait Aggression, inversed value of Social Desirability)

and Extraversion (Impulsiveness, Adventure Seeking, inversed value of Detachment). The scoring of the scale was implemented through a computerized scoring template, transforming each SSP subscale’s scores in standardized T-scores, adjusting for age, with means of 50 and standard deviations of 10[6].

Hormone analyses. 300 µL of sample material was used for the analysis. Serum proteins were precipitated with acetonitrile, and the supernatant was subjected to liquid–liquid extraction with ethylacetate–heptane on a Hamilton STAR pipetting robot (Bonaduz, Switzerland). An Acquity UPLC system (Waters, Milford, MA, USA) was used to chromatographically separate the steroids on a C-18 column (50 x 2.1 mm, 1.7 mm particle size), which was developed by gradient elution over 14 min, using water and methanol containing ammonium hydroxide as mobile phases. The UPLC system was connected to a Waters Xevo TQ-S tandem a mass spectrometer equipped with an electrospray ionization source, and the steroids were detected in negative (estradiol) or positive ion (progesterone, testosterone and cortisol) MRM mode. Two product ions were monitored for each compound to check for interferences. Analytical sensitivity and precision were determined as lower limit of detection (LLQ) and total coefficient of variation (CVs), respectively, for estradiol (3.6 pmol/L and 10.0%), progesterone (0.21 nmol/L and 8.9%), testosterone (0.11 nmol/L and 4.5%), and cortisol (0.59 nmol/L and 3.8%).

Task description. Prior to scanning, the participants received oral instructions about the paradigm. They were told that they would play a game where the goal is to score as many points as possible. During the PSAP, participants had to press one of three buttons (Option 1, 2 and 3) a given number of times in order to achieve a particular outcome. Thus, pressing 100 consecutive times the button for Option 1 resulted in the participant earning 1 point, pressing 10 consecutive times the button for Option 2 resulted in the virtual opponent having a point taken away (aggressive response), and pressing 10 consecutive times the button for Option 3 briefly protected the participant from having points stolen (protective response) (Fig S1A).

Participants responded via a three-finger button-box on the right hand and had to complete a started option before choosing a new one (Celeritas®, Fiber Optic Response System, Psychology Software Tools, Pittsburgh, PA). Options 1, 2 and 3 corresponded to the index, middle and ring finger keys, respectively. The status of the game, including total score, presses and options were projected onto a screen viewed by the participant while lying in the scanner (Fig. S1B). When the press counter reached 100, participants were informed about earning a point through black flashing positive symbols (“+”) and the subsequent total score increased by one point (Fig. S1C). Similarly, red flashing negative symbols (“-”) indicated when points were stolen from the participant, and the total score was decreased by one point (Fig. S1D). If participants were not using Option 2 or Option 3 during the task, they were provoked by having points stolen every 6-60 s. Provocations occurred immediately if participants did not use Option 2 or 3 for 5 min. Completing Option 2 or 3 initiated a provocation-free interval of maximum 60 s. Nonetheless, participants were only aware of the protective effect of Option 3. Participants were informed that they could not keep the points stolen from the opponent while the opponent was allowed to keep the points stolen from the participant.

Thus, Option 2 represents aggressive behavior without direct monetary reward. Behavioral output variables were: the total number of points won during the task, the total number of provocations received and the number of Option 1, Option 2 and Option 3 presses. In addition, a “PSAP aggression” score was defined as [1000 × No. Option 2]/[No. of total button presses × No. of provocations], as previously described [7]. While in the scanner, participants completed a one-minute trial session immediately before playing one 12-minute session of the PSAP, as described in [8]. The paradigm was programmed using E-prime® v2.0 (Psychological Software Tools, Pittsburgh, PA).

MRI Acquisition parameters. Structural and functional scans were acquired on a 3.0 Tesla whole-body scanner (Achieva dStream, Philips Medical Systems, Best, The Netherlands) equipped with a 32-channel head coil. For blood oxygen dependent level (BOLD) fMRI, 240 whole-brain dynamic scans were acquired using a T2*-weighted gradient echo-planar imaging (EPI) sequence with the following parameters: repetition time (TR) = 3000 ms, echo time (TE) = 30 ms, flip angle = 90°, 100 x 97 matrix size, 43 slices, slice thickness= 2.8 mm, acquisition time = 12:11 min. Resulting images have a 1.88 x 1.88 x 2.8 mm^3^ voxel size. T1-weighted whole-brain scans were acquired using a MPRAGE sequence with the following parameters: TR = 8.3 ms, TE = 3.8 ms, flip angle = 8°, 256 x 256 matrix size, 220 slices, slice thickness = 1 mm, acquisition time = 3:50 min. Resulting images have a 0.94 x 0.94 x 1 mm^3^ voxel size.

First level analysis pipeline. Regarding fMRI data, first-level analysis of BOLD signal change was performed by modelling the following conditions: Monetary Response (the first ten seconds of Option1), Aggressive Response (duration of Option2), Protective Response (duration of Option 3), Winning Reward (event, at end of Option 1), Stealing Reward (event, at end of Option 2) and Provocation (event, at time of provocation) (Figure S1A). Single-subject design matrices including the six head movement regressors obtained during realignment were estimated using a general linear model to determine condition-specific BOLD responses, by convolving the following task regressors with the canonical HRF plus time-derivative: Monetary Response was used as “baseline condition” and the following contrasts were computed: provocation event > monetary response, aggressive response > monetary response, winning reward > monetary response, stealing reward > monetary response, and protective response > monetary response. The first 10 seconds of Option1 were used as “baseline condition” to limit potential reward-related brain responses, as previously described in [7]. If a provocation occurred during this period, the time from beginning of Option1 until the provocation started was used.

Main task effects analyses. Single-subject contrast maps were then taken onto second level group-analysis to examine task-related effects both over the whole group, using one-sample t-tests. An initial voxel-wise threshold of *p* = 0.001, uncorrected with a standard FWE-corrected extent threshold of p = 0.05 [9].

Between-group differences analyses. Task-related brain activation across conditions (i.g Provocation > Monetary Response, Aggressive Response > Monetary Response, Protective Response > Monetary Response, Winning Reward > Monetary Response, and Stealing Reward > Monetary Response) was compared between treatments (SPRM vs. placebo) by use of two-sample t-tests to investigate the effect of treatment on BOLD reactivity. Non-parametric analyses were run on Statistical NonParametric Mapping (SnPM) 13.1.06 to confirm the results. Plots were built on SPSS Statistics for Windows, version 26 (SPSS Inc., Chicago, Ill., USA).

Voxel-wise interaction analyses. To assess the relationship between ROIs BOLD reactivity and task behavior, symptom severity, and psychometrics, we investigated the interaction effects of these variables by group on BOLD reactivity. For this purpose, the conventional two-sample t-test factorial design procedure was implemented in SPM12, with an interaction term between treatment group and task behavior (button presses), symptom severity, or psychometrics. One model was defined for each covariate of interest (aggressive responses, protective responses, DRSP irritability, AQ-RSV) models for each ROI. In the models that evaluated the relationship between brain reactivity and task behavior, the number of provocations and number of total presses were additionally included as covariates in the analyses to control for differences in variance due to differences in the number of provocations and total button presses. To assess whether personality traits influence treatment-dependent BOLD reactivity as a function of treatment, two way ANOVAs were specified on SPM12, with Neuroticism, and Aggressiveness SSP factors as within subject’s variables, and treatment group as between-group variable. Regarding symptoms severity, we focused on irritability because of its direct relationship to aggression, and on the personality factors of aggressiveness and neuroticism because previous association between neuroticism and PMDD negative affect[10].

Post-hoc analyses. The relationships between task behavior, symptoms severity, and psychometrics, and BOLD response in the aggressive response condition were assessed using partial correlations in each group separately. The models were implemented in each group separately. Number of provocations, along with total button presses were controlled for in the analyses regarding task behavior.

Supplementary figures


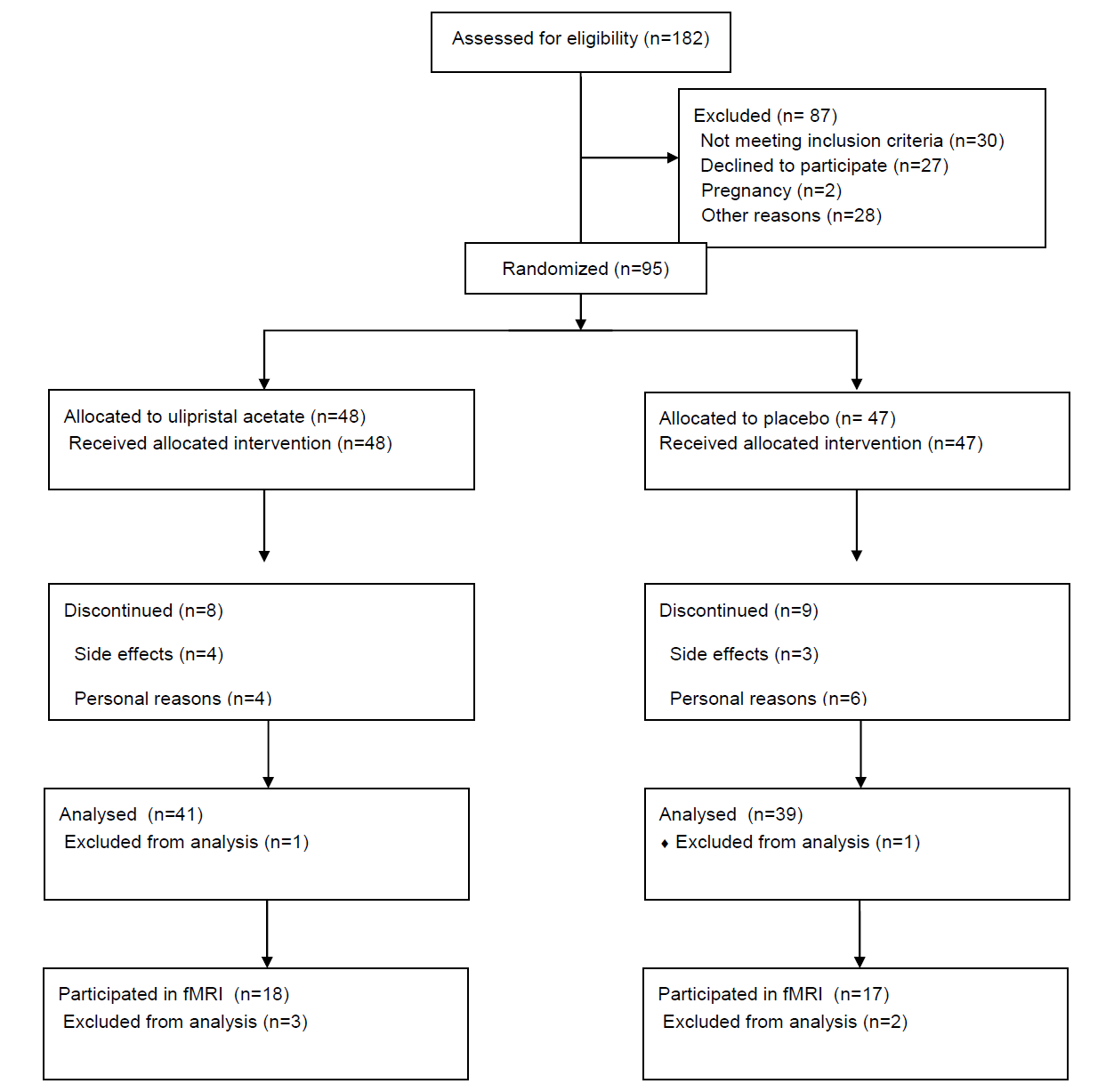


Fig. S1. Flowchart representing the clinical trial’s [5] sample and current subsample’s characteristics.


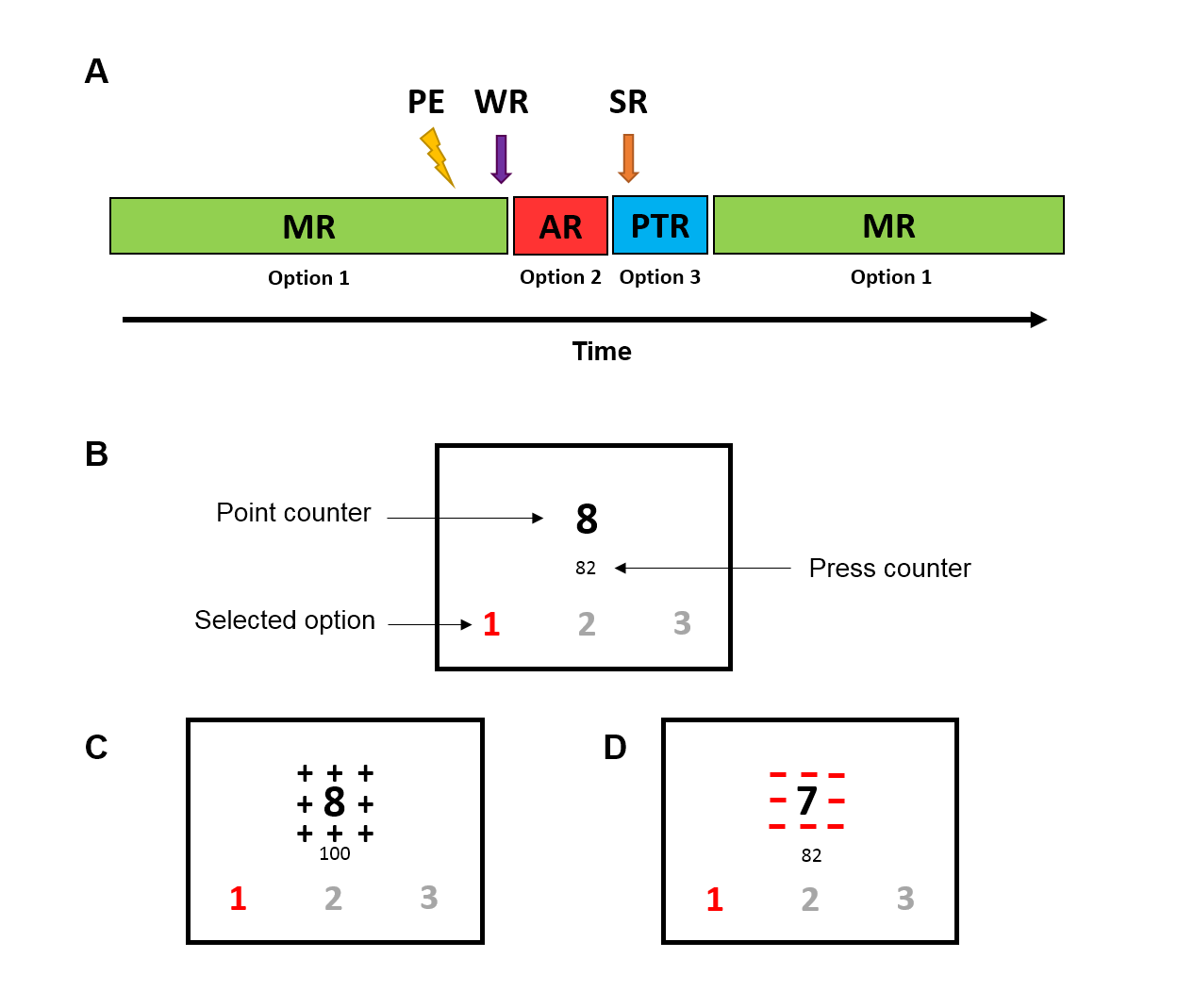


Fig. S2. (A) The bar represents the conditions and events illustrated either as blocks or arrows: MR: Monetary Response, AR: Aggressive Response, PTR: Protective Response. The ligthning icon (PE) represents the provocation event, the purple arrow (WR) the Winning Reward and the orange arrow (SR) the stealing reward. [7]. (B) Illustrative examples of what seen by the participant on the screen. (C) End of monetary response block, indicating points earned. (D) Provocation screen, indicating point stolen with flashing red icons, and the remaining points.

Supplementary tables

Table S1. Regions of interest displaying main task-effects of BOLD reactivity on the whole group, presented in MNI coordinates (mm), FWE corrected at cluster level. Cluster coordinates are presented in bold and cluster local maxima in non-bold. Activations are indicated with red and deactivations with blue. R: right hemisphere clusters, L: left hemisphere clusters.

|  | | MNI coordinates for peak voxel | | | |  |
| --- | --- | --- | --- | --- | --- | --- |
| Anatomical region | Cluster size | *x* | *y* | *z* | *T*-score | p*_FWE_*-cluster |
| Prefrontal cortex |  |  |  |  |  |  |
| *Provocation* |  |  |  |  |  |  |
| 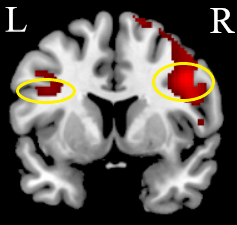 | 3176  460 | 44  42  48  -44  -48 | 8  24  12  6  26 | 34  22  24  36  32 | 8.18  5.07 | 1.35E-12  0.001 |
|  |  |  |  |  |  |  |
| 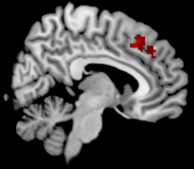 | 289 | 6  6 | 24  36 | 48  36 | 6.23 | 0.006 |
| *Winning reward*  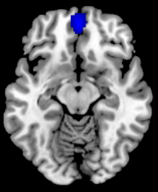 | 620 | 0  2  2 | 52  62  62 | -12  6  24 | 5.89 | 0.002 |
| Insula |  |  |  |  |  |  |
| *Provocation*  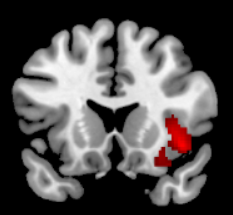 | 477 | 42  28  32 | 18  22  14 | -8  -12  -20 | 7.35 | 2.27E-05 |
| Amygdala |  |  |  |  |  |  |
| *Provocation*  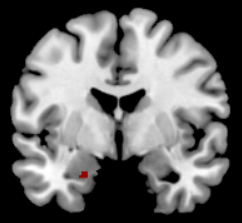 | 10 | -26 | 2 | -24 | 3.59 | 0.030 |
| Striatum |  |  |  |  |  |  |
| *Provocation* |  |  |  |  |  |  |
| 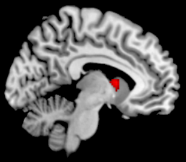 | 63 | -8 | 8 | -8 | 5.93 | 0.023 |

Table S2. Regions of interest displaying main task-effects of BOLD reactivity by group, presented in MNI coordinates (mm), FWE corrected at cluster level. Activations are indicated with red and deactivations with blue.

|  |  | MNI coordinates for peak voxel | | |  |
| --- | --- | --- | --- | --- | --- |
| Anatomical region | Cluster size | *x* | *y* | *x* | *T*-score |
| Prefrontal cortex |  |  |  |  |  |
| *Provocation, SPRM* 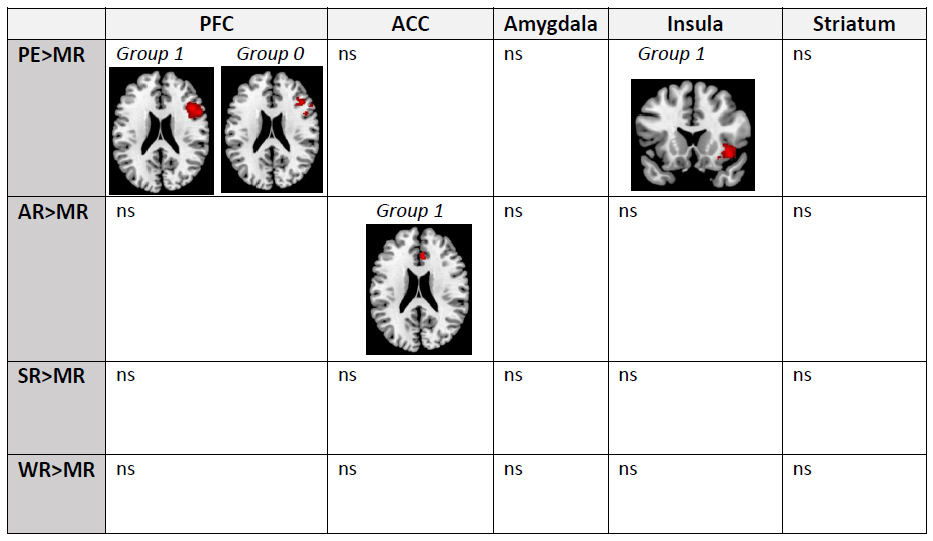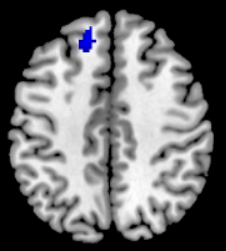 | 1423  114 | 44  -12 | 20  34 | 22  46 | 4.64  4.24 |
| *Provocation, Placebo*  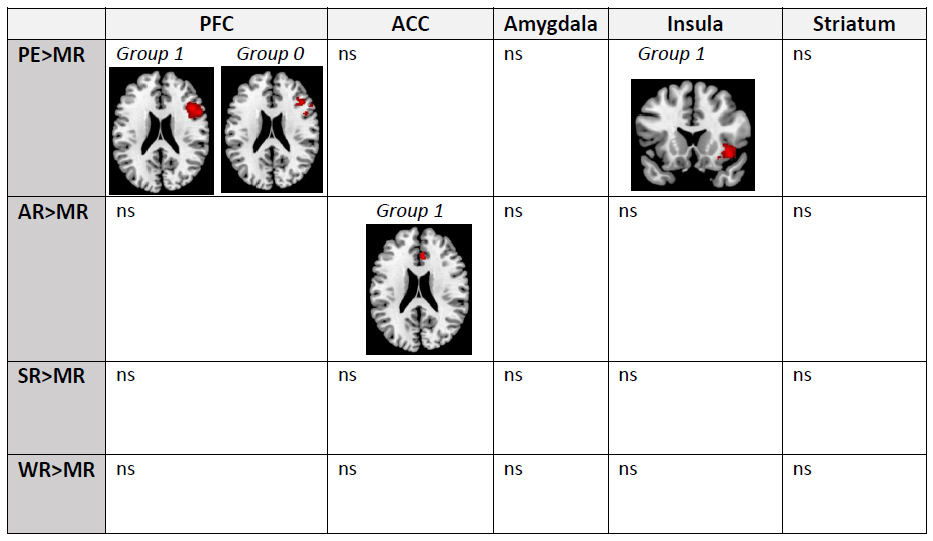 | 506 | 44 | 28 | 24 | 4.31 |
| *Aggressive response, SPRM*  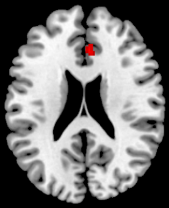 | 67 | 40 | 10 | 28 | 4.1 |
| Insula |  |  |  |  |  |
| *Provocation, SPRM*  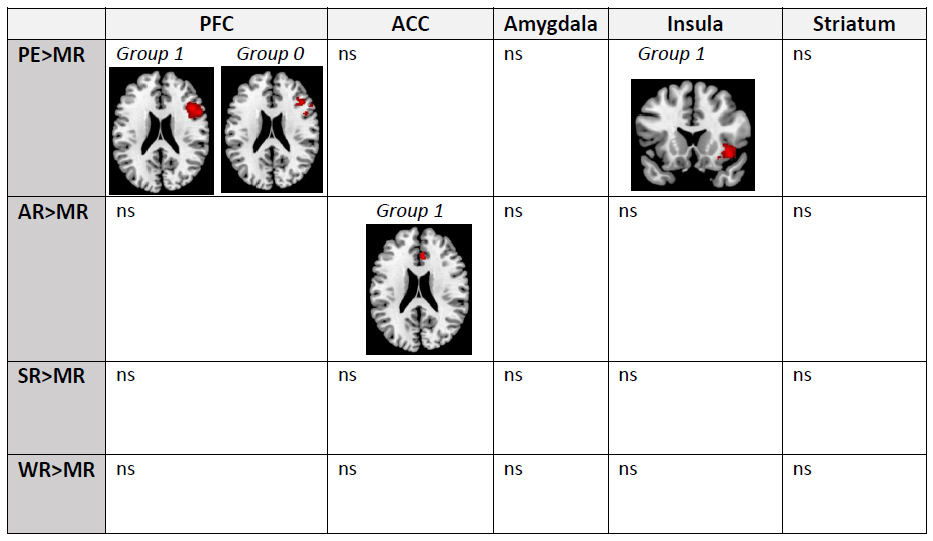 | 300 | 40 | 18 | -10 | 4.55 |
| Anterior Cingulate Cortex |  |  |  |  |  |
| *Aggressive response, SPRM*  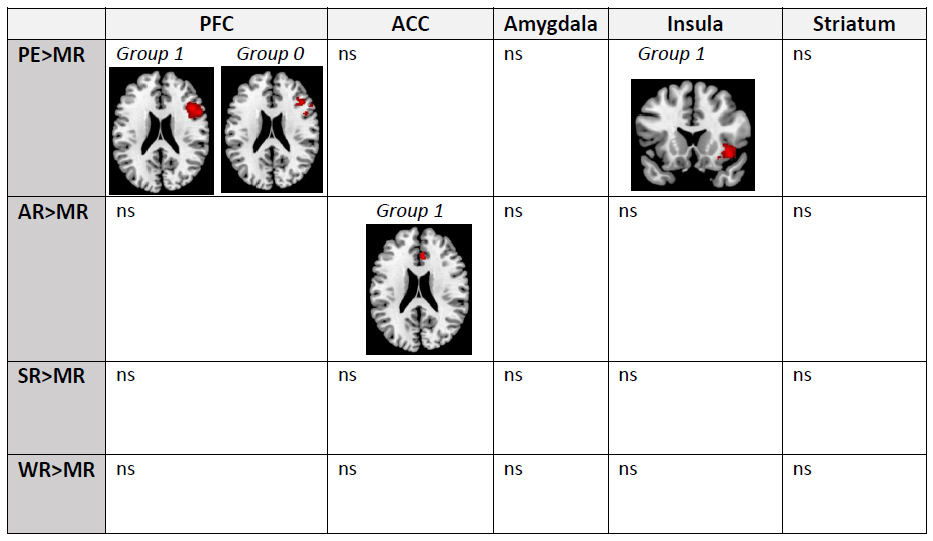 | 37 | 4 | 32 | 22 | 3.93 |
| Striatum |  |  |  |  |  |
| *Provocation, SPRM* |  |  |  |  |  |
| 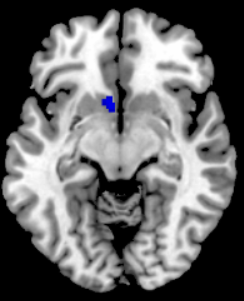 | 34 | -2 | 8 | -8 | 3.79 |

1 Eisenlohr-Moul TA, Girdler SS, Schmalenberger KM, Dawson DN, Surana P, Johnson JL, et al. Toward the reliable diagnosis of DSM-5 premenstrual dysphoric disorder: the Carolina Premenstrual Assessment Scoring System (C-PASS). American Journal of Psychiatry. 2017;174(1):51-59.

2 Yonkers KA, O'Brien PM, Eriksson E. Premenstrual syndrome. Lancet. 2008;371(9619):1200-10.

3 Whitaker LH, Williams AR, Critchley HO. Selective progesterone receptor modulators. Current Opinion in Obstetrics and Gynecology. 2014;26(4):237-42.

4 Donnez J, Tatarchuk TF, Bouchard P, Puscasiu L, Zakharenko NF, Ivanova T, et al. Ulipristal acetate versus placebo for fibroid treatment before surgery. N Engl J Med. 2012;366(5):409-20.

5 Comasco E, Kopp Kallner, H., Bixo, M., Hirschberg, A., L., Nyback, S., de Grauw, H., Epperson, C.N., Sundström-Poromaa, I. Ulipristal acetate for treatment of premenstrual dysphoric disorder – a proof-of-concept randomized controlled trial. American Journal of Psychiatry. 2020.

6 Gustavsson JP, Bergman H, Edman G, Ekselius L, Von Knorring L, Linder J. Swedish universities Scales of Personality (SSP): construction, internal consistency and normative data. Acta Psychiatrica Scandinavica. 2000;102(3):217-25.

7 Skibsted AP, Cunha-Bang SD, Carre JM, Hansen AE, Beliveau V, Knudsen GM, et al. Aggression-related brain function assessed with the Point Subtraction Aggression Paradigm in fMRI. Aggress Behav. 2017;43(6):601-10.

8 da Cunha-Bang S, Fisher PM, Hjordt LV, Perfalk E, Persson Skibsted A, Bock C, et al. Violent offenders respond to provocations with high amygdala and striatal reactivity. Social Cognitive and Affective Neuroscience. 2017;12(5):802-10.

9 Eklund A, Nichols TE, Knutsson H. Cluster failure: Why fMRI inferences for spatial extent have inflated false-positive rates. Proceedings of the National Academy of Sciences. 2016;113(28):7900.

10 Gingnell M, Comasco E, Oreland L, Fredrikson M, Sundström-Poromaa I. Neuroticism-related personality traits are related to symptom severity in patients with premenstrual dysphoric disorder and to the serotonin transporter gene-linked polymorphism 5-HTTPLPR. Archives of Women's Mental Health. 2010;13(5):417-23.
